# Exploring why a complex intervention piloted in general practices did not result in an increase in chlamydia screening and diagnosis: a qualitative evaluation using the fidelity of implementation model

**DOI:** 10.1186/s12875-017-0618-0

**Published:** 2017-03-21

**Authors:** R. Allison, D. M. Lecky, K. Town, C. Rugman, E. J. Ricketts, N. Ockendon-Powell, K. A. Folkard, J. K. Dunbar, C. A. M. McNulty

**Affiliations:** 1 Primary Care Unit, National Infection Service, Public Health England, Microbiology Dept, Gloucestershire Royal Hospital, Great Western Road, Gloucester, GL1 3NN UK; 2 HIV/STI Department, Centre for Infectious Disease Control and Surveillance, Public Health England, London, UK; 3 Formerly Public Health England, Primary Care Unit, Microbiology Dept., Gloucester, GL1 3NN UK; 4 Formerly Public Health England, Primary Care Unit, Microbiology Dept., Gloucester, now Derriford Hospital, Derriford Road, Plymouth, UK; 5 Formerly Public Health England, Primary Care Unit, Microbiology Dept., Gloucester, now Biotechnology and Biological Sciences Research Council (BBSRC), Polaris House, North Star Avenue, Swindon, UK

**Keywords:** Chlamydia, Testing, Training, Educational intervention, General practice, Implementation, Adherence, Fidelity, Qualitative, Evaluation

## Abstract

**Background:**

*Chlamydia trachomatis* (chlamydia) is the most commonly diagnosed sexually transmitted infection (STI) in England; approximately 70% of diagnoses are in sexually active young adults aged under 25. To facilitate opportunistic chlamydia screening in general practice, a complex intervention, based on a previously successful Chlamydia Intervention Randomised Trial (CIRT), was piloted in England. The modified intervention (3Cs and HIV) aimed to encourage general practice staff to routinely offer chlamydia testing to all 15–24 year olds regardless of the type of consultation. However, when the 3Cs (**c**hlamydia screening, signposting to **c**ontraceptive services, free **c**ondoms) and HIV was offered to a large number of general practitioner (GP) surgeries across England, chlamydia screening was not significantly increased. This qualitative evaluation addresses the following aims:

- Explore why the modified intervention did not increase screening across all general practices.
- Suggest recommendations for future intervention implementation.

**Methods:**

Phone interviews were carried out with 26 practice staff, at least 5 months after their initial educational workshop, exploring their opinions on the workshop and intervention implementation in the real world setting. Interview transcripts were thematically analysed and further examined using the fidelity of implementation model.

**Results:**

Participants who attended had a positive attitude towards the workshops, but attendee numbers were low. Often, the intervention content, as detailed in the educational workshops, was not adhered to: practice staff were unaware of any on-going trainer support; computer prompts were only added to the female contraception template; patients were not encouraged to complete the test immediately; complete chlamydia kits were not always readily available to the clinicians; and videos and posters were not utilised. Staff reported that financial incentives, themselves, were not a motivator; competing priorities and time were identified as major barriers.

**Conclusion:**

Not adhering to the exact intervention model may explain the lack of significant increases in chlamydia screening. To increase fidelity of implementation outside of Randomised Controlled Trial (RCT) conditions, and consequently, improve likelihood of increased screening, future public health interventions in general practices need to have: more specific action planning within the educational workshop; computer prompts added to systems and used; all staff attending the workshop; and on-going practice staff support with feedback of progress on screening and diagnosis rates fed back to all staff.

**Electronic supplementary material:**

The online version of this article (doi:10.1186/s12875-017-0618-0) contains supplementary material, which is available to authorised users.

## Background

Chlamydia trachomatis (chlamydia) is the most commonly diagnosed STI in England, with 206,774 diagnoses made in 2014 [1]. Sexually active young adults aged under 25 years old continue to be at the highest risk of contracting an STI [1]. Chlamydia infection can cause significant short and long-term morbidity with complications including: pelvic inflammatory disease (PID), tubal infertility and ectopic pregnancy in females; and epididymo-orchitis and possible infertility in males [2–4].

The National Chlamydia Screening Programme (NCSP), established in 2003, aims to control chlamydia through early detection and treatment of asymptomatic infection, so reducing onward transmission and the consequences of untreated infection [5, 6]. A means of achieving this is through opportunistic screening [7–9]. As the majority of young people visit their GP surgery at least once annually, young people see it as an accessible and acceptable setting to receive sexual health services [10–12].

To facilitate opportunistic screening, Public Health England (PHE), in consultation with a primary care led advisory group comprising of general practitioners and practice nurses, developed a more complex intervention, based on a previously successful Chlamydia Intervention Randomised Trial (CIRT) [13, 14]. Whereas the only aim of CIRT was to increase chlamydia screening, the modified intervention, 3Cs and HIV [15], incorporated updated national policy change, such as the integration of chlamydia testing with other sexual health and reproductive services. It aimed to encourage general practice staff to routinely offer chlamydia testing, and provide information about the provision of contraceptive services and free condoms (the ‘3C’s) to all 15–24 year olds, regardless of their reason for consultation. Additionally, practice staff were encouraged to offer HIV screening in line with national guidelines [16].

**Fig. 1:**
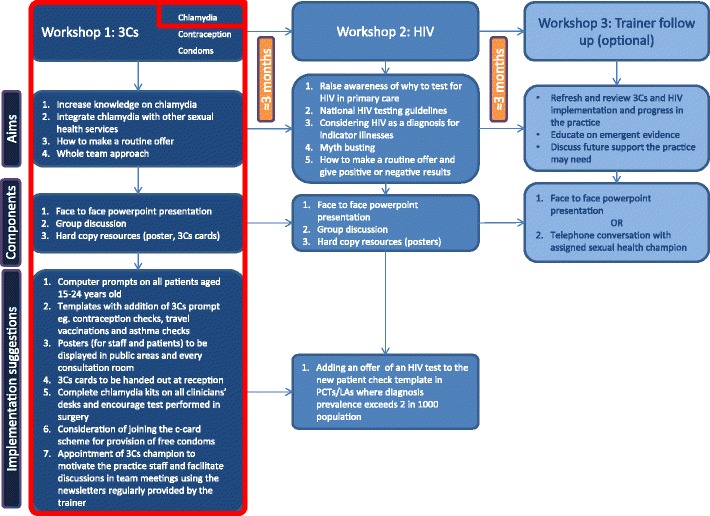
The 3Cs and HIV intervention with the components that aim to increase chlamydia screening and diagnosis rate highlighted in red

The 3Cs and HIV intervention, based on the Theory of Planned Behaviour [17], consisted of two educational workshops, delivered by trained local sexual health staff, and an optional follow up with the trainer (see Fig. 1 for details). The first workshop focussed on the routine offer of 3Cs (chlamydia, contraception and condoms); and the second on facilitating HIV screening. The workshops, delivered between August 2013 and July 2015, were supported by a range of materials to engage practice staff and patients (posters, invitation cards and/or leaflets) [18], such as: advertising the availability and confidentiality of the service, in waiting areas; as a prompt for clinicians, in consultations. These resources were developed based on the Theory of Planned Behaviour [17], with engagement in mind. The intervention encouraged a ‘whole practice’ approach to screening, encouraging all staff members (clinical and non-clinical) to attend the educational workshops; and suggested a 3Cs champion be appointed to motivate, encourage and facilitate discussions and feedback within team meetings.

**Fig. 2:**
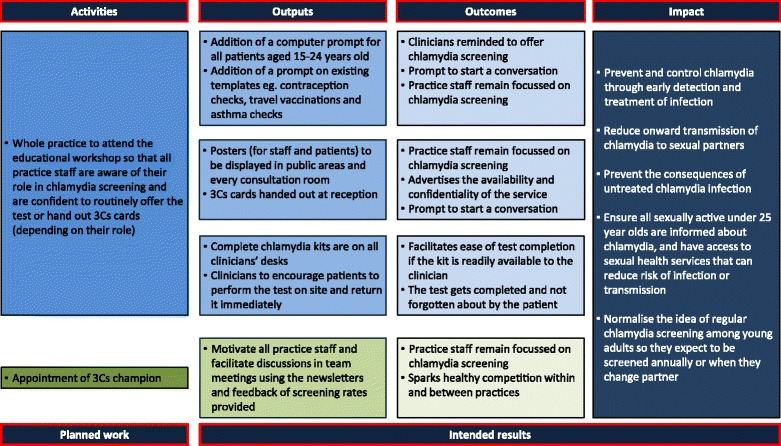
Modified logic model of how the suggested components of the intervention could have been implemented to increase chlamydia screening

Following the protocol for the 3Cs & HIV programme [15], both quantitative and qualitative methods were planned to evaluate the effectiveness of the intervention in relation to increasing chlamydia screening (see Fig. 2 for more details on how the intervention was designed to increase chlamydia screening, specifically).

Although immediately after the workshop, practice staff who attended the educational workshops fed back that they intended to increase their chlamydia screening as a result of the training, the quantitative evaluation shows that there was no overall significant increase in chlamydia testing or diagnosis across all of the practices offered the workshops [19]. However, chlamydia testing and diagnosis significantly increased in practices that received specific payments for testing before the intervention began. These practices were reminded about these incentives during the educational workshop.

This qualitative arm of the service evaluation aimed to: a) explore why an intervention based on a successful trial [13, 14] did not achieve the same sustained results in the wider pilot, b) make recommendations for future interventions, and their implementation, within the general practice setting in an effort to improve outcomes.

**Fig. 3:**
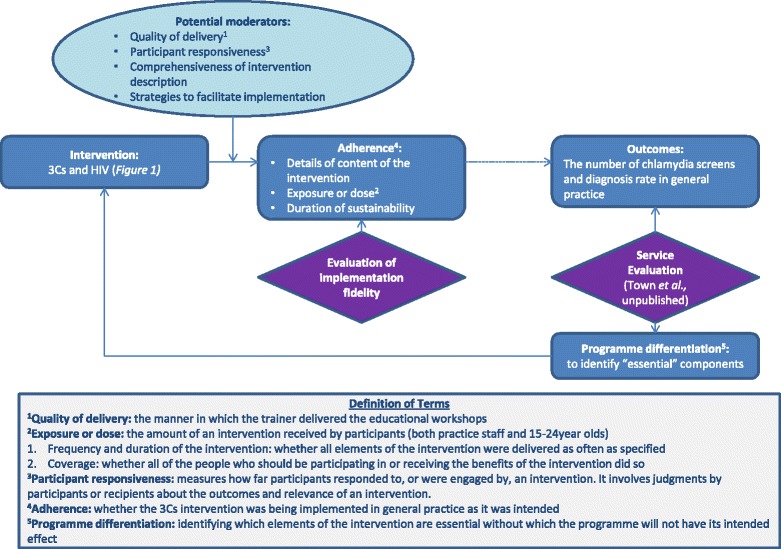
Modified conceptual framework for fidelity of implementation [20]

The fidelity of implementation model (Fig. 3) has been used to facilitate this research. The theory provides a conceptual framework referring to the degree to which an intervention or programme is delivered as intended [20]. Unless such an assessment is made, it cannot be determined whether a lack of impact is due to poor implementation or inadequacies inherent in the programme itself, so called Type III error [21, 22]. The concept of implementation fidelity can be described in terms of five elements: adherence to an intervention; exposure or dose; quality of delivery; participant responsiveness; and programme differentiation [23]. This paper will explore each of the first four elements, the relationship between them and suggest essential components of the intervention (programme differentiation, the fifth element) based on the qualitative and quantitative findings.

## Methods

### Recruitment and sampling

Practices that had participated in the 3Cs and HIV intervention were stratified by chlamydia screening rate per 100 registered 15–24 year old patients, from January 2013 until September 2014 [19]. Practices were then contacted in a random order from this list via the practice managers. Interviews were arranged with practice staff that had previously attended the educational workshops. Data saturation was achieved after 18 interviews [24]. However, as the quantitative analysis showed locally enhanced service (LES) payments to be a relevant factor [19], purposive sampling [25] was used recruit a further 8 participants from areas with an existing LES, at the time of the intervention. At this stage, male practice staff were prioritised, where there was an option, to reduce the gender imbalance.

### Interview schedule

The interview schedule explored staff’s opinions on the 3Cs and HIV educational workshop as a whole; which components of the intervention they had implemented and their opinion on these (ie. posters, cards, prompts, champion, further discussion); and the impact the intervention had had on their chlamydia testing, HIV testing and sexual health provision. Participants were specifically asked about any barriers to offering basic sexual health services in their practice and ideas on how these could be alleviated. In the initial stages of qualitative data collection, the interviewers were not aware of the quantitative findings. The semi structured interview schedule [26] was modified and developed throughout the data collection process, based on the findings from previous interviews and, following the results of the quantitative analysis, to assess why the intervention was not successful, overall (Additional file 1: Appendix 1 and Additional file 2: Appendix 2). Interviewers were practiced in the skill of interviewing, using probing as opposed to leading questions. Length of interviews ranged from 12.8 to 50.6 min, with an average length of 31.6 min.

### Interview sessions

Interviews were conducted by telephone, as practice staff reported that they had insufficient time for face-to-face interviews. Although visual cues cannot be obtained during telephone interviews [27, 28], results compared between telephone and face-to-face interviews are very similar [29]. In order for the interviewee to feel they could speak openly and not be influenced by a pre-existing relationship with the interviewer, the interviewer had not facilitated the training or had any previous contact with the participants. Therefore, in order for the interviewer to build rapport with the practice staff, emails and phone calls were exchanged, in order to: set up a time for the interview; discuss the purpose of the research; and ensure the participant was fully informed before written consent was provided. Interviewees were explicitly encouraged to discuss all positive and negative features of the intervention, in order to understand facilitators and barriers to implementation.

### Analysis

Interviews were recorded and transcribed verbatim. Transcripts were analysed using a modified framework analysis [30]. Transcripts were initially read through for accuracy and data familiarisation. Two researchers independently coded categories and themes: the lead researcher coded all transcripts and a second researcher coded 10% of the transcripts, to ensure coding consistency; minor discrepancies over specific terminology of themes were resolved through discussion and referral to the original transcripts until agreement was reached. Use of NVivo software (version 10) facilitated the organisation of the data. The one sheet of paper (OSOP) method [31] was used to clarify findings within, and between, themes. As the quantitative evaluation found that the intervention was not successful in increasing chlamydia screening and diagnosis rates in all general practices [19], deductive analysis [32] was used, whereby the principles of the fidelity of implementation model (Fig. 2) were applied to further analyse the themes.

## Results

From July 2014 to May 2016, at least 5 months post intervention, 26 members of practice staff were interviewed to see whether there had been a sustained behaviour change. Both clinical and non-clinical staff were interviewed, as everyone had been encouraged to attend the educational workshops. More women participated in the interviews than men, which is also reflected in attendance of the educational workshops. See Table 1 for more details.

**Table 1:** Characteristics of participants (*n* = 26) and practices (*n* = 19)

Participant characteristic	Variable	Number of participants
Job Role	GP	9
	Practice Nurse	13
	Practice Manager	3
	Receptionist	1
Gender	Male	5
	Female	23
Age	30–40	3
	41–50	6
	51–60	6
	Unknown or did not wish to declare	11
Treating young people or providing sexual health services	None	7
	Some	6
	Heavily involved	13
Practice characteristic	Variable	Number of participants
Local Authority	Devon	3
	Kingston	4
	Luton	2
	North Somerset	5
	York and North Yorkshire	10
	Lincolnshire	2
Phase of implementation (stepped wedge design) [15]	1	4
	2	19
	3	3
Enhanced service	Yes	14
	No	8
	Unknown	4
Chlamydia testing rate higher than England median (pre-intervention)	Yes	19
	No	7
Number of GPs employed	2–5	8
	6–10	5
	11–15	4
	16+	9

The themes that emerged from the interviews were further examined using the fidelity of implementation model as outlined below.

### Quality of delivery

#### Opinion on the trainer/training

Practice staff were very positive about the trainer, quality and format of the training received.

“actually quite interested to hear all the statistics because she did say a lot about the (*geographical location*) area and what was relevant to us rather than just about the whole country” (KIKT122- receptionist)

“The advantages of training like the 3C are that staff can talk to each other at in group sessions, and any sort of group training is good because, people, not from what you learn particularly from the lecturers, but from what you learn from the colleagues” (YNYCR126- nurse)

Participants felt that it was relevant to all staff, regardless of their previous sexual health training or their current role within the practice.

“I found it very useful, and it was… delivered at the right level… we had admin staff with varying knowledge and years of experience working in the NHS as well as two clinical staff attend. So she pitched it at the right level, made it interesting, made it informative and yeah, she’s a very pleasant lady and I think she did very well… Yes, no complaints whatsoever. It was easy to understand. There wasn’t any massive medical jargon. It gave a bit of background history as to each point and yeah, she delivered it well.” (DRA2- practice manager)

The training raised staff awareness of chlamydia and was facilitated by the fact that the trainer came to the practice and held the training over lunch, so it was not an added burden to staff’s workload. In a couple of practices, the 3Cs and HIV educational workshop prompted individuals within the practice with an interest in sexual health to provide further training sessions for staff.

“I think it’s made people more aware, because what we did was, we also expanded on the contraceptive side of things and we actually gave a very short training session to the staff as to the types of contraceptive that we actually offer…” (DRA2- practice manager)

#### Suggestions for the future

Most staff said that they did not: receive the 3Cs and HIV newsletter provided by the trainer to the practice for dissemination; or any other feedback on their progress; and were not aware of any further contact with the trainer following the start of the intervention.

“Never, ever had any feedback after the training…The training was very good, it was comprehensive, but I was asked then, to produce these figures and I’ll never know what they were for.” (DRA2- practice manager)

“I don’t think we got any support from my memory, I can’t remember having anything…which I think would have been helpful” (NSCR122- GP)

Participants recommended regular updates circulated to all staff, as initially staff were motivated and focussed, but as time passed, this was not sustained.

“We did have quite a big drive originally, and things just tend to drop off sometimes…

What do you think could be changed so that it doesn’t drop off?

I think just keeping up with this kind of training, really, and keeping us on our toes, I suppose…. we just need to do the reminders, I think, more than anything else.” (LIRA2- nurse)

Suggested formats included: the trainer coming back to train new staff; shorter but more regular training slots; making the training mandatory; training for a group of GP surgeries rather than individual surgeries; more regular updates on STIs.

“…you do a big screening event, for the few weeks after the event, it’s foremost in everyone’s mind because they’ve just had the training. A year later, they’ve forgotten all about it… If the same person that delivered the first training turned up 6 months later and said I’m only here for 10 minutes, do you remember we did the screening, the training, how are you getting on? Have any anything you want to discuss about difficulties?… You know if you just had a 10 minute refresher every 6 months that would, that would keep the plant watered” (NSCR121- GP)

“I think probably tiny amounts of training on a on a frequent basis…may be better than a big training session which nobody has got time for… I think small but regular training inputs are more effective” (NSCR121- GP)

“**Following the training**, **were you clear on what your role was in taking the 3Cs forward and offering a routine chlamydia test**?

At the time I was. I think I was crystal clear. Yeah. I’d like, I wouldn’t say I am now.

**And why do you think it is**?

…I need things repeating to me every so often and I think that’s what’s missing. I think if we had something like the 3Cs training yearly, that would keep it fresh in my mind… Yeah we do a lot of things yearly. It’s mandatory for a lot of things and that’s how we remember it. Because we’re all rubbish at remembering… So it would fit with something like that.” (YORA4- GP)

“The GPs have training every month… and that is the best way to get to GPs, because they’re all there at the same time… there’s nurse training as well, that we have every month.” (YORA1- nurse)

### Exposure or dose

#### Who attended the training vs. who the training would be most useful for

Data from the quantitative analysis showed that 58% (268/460) of practices that agreed to participate in the intervention received at least one educational workshop [19]. When discussing reasons for their colleagues non-attendance at the educational workshops, participants reported reasons, such as: lack of time (especially GPs); staff feeling that it is not their field, role, or area of expertise (especially male, older GPs); and the perception that chlamydia tests are offered in contraception consultations, which are generally for female patients, who prefer to see female staff.

“We didn’t complete it… only did the chlamydia and it’s not because she didn’t try, she kept trying to get in, but we had a massive, massive staff change round and then we’ve had a lot of sickness so I couldn’t, I just cannot get the meetings together for the rest of them.” (YNYER121- nurse)

“you’re going to find that female staff will go… it’s also in your area of speciality and it’s going to be interesting whereas, yeah, someone who’s just not got that in their kind of bag is not, unless it’s a learning need for them, they’re probably not going to rush to be there… are going to miss training” (DCR120- GP)

“[male GP talking about patient’s preference]…see the female doctors for, for consultations… I think it’s less of an issue less of something I’ve had to deal with than some of my colleagues but that of course puts me at risk of being less good at it” (NSKT124- GP)

Some participants felt that the training would be most beneficial for the staff that do not deal with sexual health or young people on a regular basis, but it was felt that these were often the members of staff that did not attend the training.

“If we didn’t already have the experience of talking to young people because we are a university eh campus site then I think that those for those clinicians then it would be hugely beneficial” (YNYKT125- nurse)

### Staff’s perception of young people’s beliefs

It was felt that 15–24 years olds are overall more aware and responsive to the offer of a chlamydia screen than previously, due to campaigns related to screening, and education in personal, social, health and economic education (PSHE) and sex and relationship education (SRE) in schools. As such, participants reported that chlamydia screening has become more routine.

“Yeah, they are more aware and a lot of them have had, yeah a, well a lot of them say that they have had chlamydia screening in the last 3 months, when I’ve asked them, and they’re aware to do it every year if they’ve got a regular partner or when they change their partner” (NSCR122- GP)

“It’s part of their, you know, obligatory PHSE training in school now, they all have a lot of time erm being taught that, so I think, perhaps, patients don’t feel the need to come in and be taught that in the surgery, because they’ve already had it” (NSCR120- GP)

Practice staff reported that the 3Cs were mainly offered to young women in contraception consultations, as there was a reminder on the contraception template. It was the opinion of some practice staff that if a patient wanted to be tested for chlamydia, they would just pick up a self-screening kit, and therefore, did not need to be offered a test.

“I haven’t had the personal experience that that many people are coming in asking for screening. I think, if they want screening, they know they can pick up the self-testing kits” (NSCR120- GP)

It was the opinion of many practice staff that young people have a preference for receiving sexual health advice from services other than general practices. Reasons included: lack of GP appointments at appropriate times; preference to be in an environment with people of similar age to them; and the worry about the lack of confidentiality in general practice, especially if practice staff know the patient’s family members. Several sources gave their opinion that young people would rather attend sexual health or GUM clinics to get advice on sexual health or pick up a chlamydia screening kit.

“But for the young, with the time, they might talk about after school, before school, and general practice is quite restricted in time.” (LUCR120- nurse)

“They like that so and I think they feel more comfortable about accessing that service if there is people of a similar age to them” (KICR123- nurse)

“Young people that, they like to go somewhere where they think no one knows them and they’re worried maybe if they know the receptionist, or someone’s going to tell their parents, and they don’t realise how confidential the service is really, so that’s why I think they like anonymity of going maybe to a CASH service or an integrated contraception and sexual health service” (KICR123- nurse)

### Participant responsiveness

#### Practice staff’s responsiveness

**Whose role is sexual health**?

There were differing perceptions from participants on whose role sexual health was and whose responsibility it was to implement the suggested changes of the intervention.

“[talking about their colleagues’ perceptions] Not their field not their problem. Those sorts of patients go to family planning or women’s health or the nurses” (DCR120- GP)

This lack of clarity often resulted in components of the intervention not being completed, as individuals expected someone else to be doing it.

“**Do you know whether**, **following the training**, **you had a sexual health champion or someone that reminded**, **kept prodding you to**

Do you know what? We didn’t. That would have been a good idea. We didn’t. I think we rely on Dr ** in the practice and Dr ** [female GPs], an awful lot with this. But I think we haven’t actually got a champion.

**Do you know whether that was something that was discussed or within the training**?

Do you know, I think it was. I think actually in the meeting afterwards I think it was, and for my knowledge I don’t know if we’ve got one. But there could be, well be one in place and I’m just not aware of it.” (YORA4- GP)

“Doctors will be aware of it but they just I suppose haven’t got the time and they just think that nurse, the nurses are doing it [laughs] they just think the nurses are doing it” (KICR123- nurse)

Participants also reported a lack of discussion amongst staff members about chlamydia testing, especially between clinical and non-clinical staff.

“Probably if we had a meeting with the GPs and had a discussion about it and as to what the policy should be, that would probably be more useful, then we’d know where we stand really, If the GPs were on board with offering it then we would if, we’d probably do it ourselves more often as well” (YNYCR122- nurse)

There was a mixed response from non-clinical staff on their willingness to participate in the implementation, for example, some reception staff felt uncomfortable handing out the 3Cs invitation cards to patients.

“Because I kind of did all the spiel and left all the kits at reception and various information, only to discover when I checked a little while later if they’d been offering it, they said, ‘oh, well no one’s asked’” (KICR123- nurse)

“It’s not considered our role to ask patients if they want to have chlamydia screening done or anything like that…if someone wants a chlamydia test we’d book them in with Nurse C*” (KIKT122- receptionist)

### Patient responsiveness

The majority of staff felt that young people are receptive to a discussion about 3Cs.

“Where we work, there’s decent awareness of chlamydia amongst young people as an issue, so provided its phrased sensitively, I don’t think there’s a particular issue with making that offer” (NSKT124- GP)

Some participants stated that young people expect to be offered 3Cs as they are in the target age range. It was highlighted that the best approach was to phrase the offer of 3Cs correctly; emphasising the routine, non-judgemental nature of the offer. Gender difference in response to the offer was not identified, but it was highlighted that young men are especially hard to target as they did not attend as frequently.

“I mean men of that age, boys don’t, if they’re not coming for contraception they don’t consult… and when they do consult they’re not consulting about anything to do with sexual health… so for men, you’ve got somebody coming in about their asthma, and then you’re saying to them, as the doctor, ‘while you’re here, do you want a chlamydia check?’ Its, they’re going to look at you, thinking, ‘I’ve come about my asthma, why are you suggesting I need sexual, you know, why are you suggesting I’ve got a sexually transmitted disease?’” (NSCR121- GP)

In contrast, young women were often offered sexual health advice and chlamydia testing at contraception check-ups, as there was usually a tick box for this on the contraception computer template. Some sources said that it was more difficult to offer the 3Cs if the young person was accompanied. Despite the educational workshops, some sources still perceived that patients responded negatively to an offer of a chlamydia test, citing reasons, such as: the perception that patients are offended and feel judged if offered in a non- sexual health consultation; and that there is an excess of chlamydia testing offers to this age group, which causes frustration.

“And there are, some youngsters are constantly being offered chlamydia tests. They get offered them at college, they get offered them every time they go into the toilet there’s a poster about it and if you’re just unlucky enough to be the seventh person that week that’s offered them a test…they can actually be slightly irritated that it’s being overdone” (NSCR121- GP)

### The difference between wanting to implement change and making that behaviour change

A locally enhanced service, including financial incentives was mentioned as a facilitator that could make a small difference, but targets set to gain these payments were often reported by participants as unrealistically high. One interviewee highlighted that offering the 3Cs becomes easier, more routine and more comfortable with practice.

### Adherence to an intervention

#### Practice adherence: prompts and templates

Most staff said that they now had a reminder to offer a chlamydia test on the contraception template, which was useful, but no practices had added it to any other consultation template.

“The contraception template, there’s, you know, a tick box for condom supplied, tick box for chlamydia discussed or offered, and tick box for contraception counselling. So, you know, it does kind of, if you use the template, it does trigger you to go through those three things.” (DCR120- GP)

“…prompts on our, just for contraception about screening for chlamydia. We don’t have any other prompts” (NSCR122- GP)

Very few had added a computer prompt based solely on the age of the patient, which would encourage offering a chlamydia test to anyone in the age range, not just females presenting for their contraception consultations.

“I don’t think we could manage to make it toggle into our template, so I think then, it’s a separate thing we have to search for, and I think that is probably something that gets missed” (DCR120- GP)

#### Practice adherence: hard copy resources

All of the practices interviewed had made use of the posters and 3Cs cards provided in the 3Cs and HIV intervention. Many had positive views on the resources, reporting that they acted as good marketing tools to advertise the chlamydia screening service and highlight its confidential nature.

“We’ve got notices up saying that it’s about confidentiality… And I think, actually, it’s really useful having the posters because you can say, ‘this is a national screening programme.’” (NSCR120- GP)

The majority displayed the posters in the waiting rooms or a prominent place in corridors.

“We actually set up a campaign in the waiting rooms… to say that we were going to ask about chlamydia” (YNYER121- nurse)

Very few displayed the posters in clinical rooms, but those that did said that it was a useful prompt to open the conversation, as suggested in the intervention training videos used in some educational workshops. 3Cs cards were mainly left on reception or in the waiting area for patients to pick up, however, interviewees often did not know whether the receptionists had handed these out, as follow up discussions between practice staff about how the intervention had been implemented had not occurred.

### Practice adherence: sexual health champion

Although it was suggested in the educational workshops, some practices were unaware of whether they had a sexual health champion; and when appointed, a couple of sexual health champions were unclear of the requirements of their role.

“I think we just need an update, and a reminder that we need to be thinking, that involves more of our staff. I’m sure, if somebody came, they wouldn’t tell us anything more than we already know, but sometimes you need that prompt every now and again.

**Was there anyone within the practice that was giving those prompts**?

No, I probably should do it as lead, but again, you just, it just gets absorbed into everything else that you’ve got to do.” (YORA1- nurse)

### Barriers to adherence

Competing priorities; lack of time as patients often come in with more than one issue to discuss; or forgetting to offer the 3Cs, especially in a non-sexual health consultation, appeared to be the main barriers for practice staff.

“If a young person has come to talk about something else, it’s not always appropriate to then start talking about 3Cs…sometimes they kind of come with a shopping list of things that they want to come and get dealt with in 10 min, which is all, all medical, and then you are then going to add in well let’s talk about 3Cs, and if you do that properly, that really… can be a 10 min consultation itself, because it opens can often open up a whole can of worms and a whole other consultation and discussion with the patient, just, just to talk about the 3Cs” (YNYKT125- nurse)

“If a young man presents with back pain or shoulder pain then I wouldn’t really, I’m not very good at remembering” (NSCR122- GP)

However, a couple of participants pointed out that, although offering the 3Cs may mean a little extra work, it is something they are meant to, or should routinely be doing.

“**So has working in a way**, **offering the 3Cs**, **meant that you**’**ve got any more work to do**?

No… It’s just made me do the work that I was meant to do.” (YORA4- GP)

Lack of complete chlamydia kits or condoms in clinicians’ rooms were also limiting factors.

“Our support staff don’t have the kits made up…and that has hindered it… you know, if each time, I’ve got to go and find it, sort it out myself… and you look a plonker, you look a right wally. You know, if you say, ‘oh there’s a kit in the waiting room go’ and there isn’t… it’s down to couple of key individuals that just they perhaps see their role as that.” (NSKT123- GP)

“It’s all very well for me to sit here running 20 min late and think and four people in the waiting room waiting for me, and to think, ‘oh, I should offer this person chlamydia screening’. But, unless that’s at my fingertips, in terms of information for the patient all the testing materials… it makes me think, ‘oh I just can’t I just can’t do that now.’” (NSKT124- GP)

Lack of privacy in the reception area and lack of opportunity proved the main barriers for receptionists handing out the 3Cs cards.

“Because, if you’re a receptionist who’s not, not a clinician, it’s difficult to ask. You can’t. How can you ask someone, in the reception area, with a queue of people?” (NSCR120- GP)

Due to time pressures or lack of facilities, not all practices viewed the video clips as part of their educational workshop. These videos suggested “scripts” that could be used in different consultations to facilitate the offer of 3Cs.

## Discussion

### Summary

Although participants were very positive about the educational workshops, they reported that not all staff attended and, therefore, a whole practice approach was not attained. Some practices did not plan who was going to take the intervention components forward and how this would be done. For example, patients often did not complete the test immediately and the main reason given was that kits often were not made up and in the clinicians’ rooms.

Additionally, although the educational workshops specifically encouraged the use of computer prompts and templates, practices generally only added a 3Cs prompt to the female contraception template. Many practices did not develop automatic computer prompts for all patients aged 15–24 years old; therefore, men were excluded [4, 33]. Nevertheless, if all patients attending their GPs for a contraception consultation had completed a chlamydia screen, the intervention effect would have been greater than observed [34], suggesting that this did not happen.

Furthermore, although the educational workshop explicitly addressed some misconceptions of patients’ preferences, practice staff’s opinions and beliefs had not always changed. This is highlighted in the opinion of a few practice staff that patients would just pick up a self-testing kit if they wanted to be screened; although the point of the intervention was to routinely offer a chlamydia screen to all 15–24 years olds, even if un-symptomatic and in un-related consultations. Although screening may be available through other services, the evidence provided at the educational workshops showed that young people see the GP setting as a non-threatening place for them and therefore an appropriate setting to be offered chlamydia screening [10]. Many participants stated that, as chlamydia screening is becoming more routine, many patients had already completed a test recently and therefore refused the offer. It should be acknowledged that the number of times practice staff offered the test was not reported in the quantitative results; only the number of complete chlamydia screens. It may be that, as chlamydia screening kits are more readily available, young people are getting screened through other means, but as data shows that 60–70% of young people in the target age group visit the GP surgery at least annually [6], practice staff should still be encouraged to offer chlamydia screening to 15–24 year olds, routinely.

Practice staff were not aware of any on-going support with the trainer, which is a key difference from the successful CIRT trial; and, although trainers provided regular newsletters, these were not always circulated around the whole practice [13, 14]. Furthermore, in CIRT, the trainer was funded from a research grant; whereas the 3Cs & HIV intervention used existing staff resources to see whether the intervention was achievable and sustainable in a real world setting. The results suggest there are many challenges with moving from an RCT to real world setting.

Competing priorities, and time were the main barriers for implementation [35]. These competing priorities were: within the general practice, overall; within a consultation, when the patient came in with many symptoms to discuss. Staff reported that financial incentives themselves were not a motivator as targets were too hard to achieve, however the quantitative results show that the practices with financial incentives had a significant increase in chlamydia screening and diagnosis [19]. It is possible that the presence of LES agreements is an indicator of a pre-existing interest in the topic.

### Strengths and limitations

Complimenting a quantitative service evaluation with a qualitative evaluation can achieve various aims, including: corroborating findings; generating more complete data; and using results from one method to enhance insights attained with the complementary method [36, 37]. This paper utilised a qualitative approach to attempt to explain and provide insights into the quantitative results. The qualitative findings reflect real issues with any intervention implementation in primary care [38, 39] and the approach enables understanding of ways to improve interventions to facilitate better implementation of already proven effective complex intervention strategies on larger scales outside of trial conditions, with existing local authority funded staff [40, 41].

An additional strength of the study is the range of staff, and characteristics of practices, interviewed. For example, a mixture of male and female: GPs, nurses, practice managers and receptionists; from a range of sized practices with or without a LES, were interviewed. Furthermore, data saturation was reached and no new themes emerged, confirming rich data and a rigorous methodology.

A further strength includes the consideration of the role of the interviewers. The interviewers had not delivered the workshop to the participants and therefore had not developed a relationship with the participants around the intervention delivery. However, the interviewers had been involved in intervention development and/or analysis and therefore had a good understanding of how the intervention was intended to be delivered and the reasoning behind this. This allowed for informed probing by the interviewer, but the participant was not affected by social desirability bias [42] as they did not link the intervention and the interviewer. The primary analyst had not been involved in the intervention development, and therefore was not biased in their analysis.

As this was a service evaluation across England, it was not possible to video the educational workshops to ascertain whether they were delivered as intended. Trainers were encouraged to adapt the presentation to suit their own presentation style and add statistics to make it applicable to the local authority. Therefore, it cannot be confirmed whether the presentation was delivered exactly as intended and whether the key messages were relayed in their entirety. This highlights the difficulty in ensuring a high fidelity of delivery when working with a wide range of people in differing contexts. For example, trainers reported that, sometimes, there were not the facilities to play the video clips role-playing typical situations, and occasionally there was no training room, so the educational workshop was delivered in the waiting room with staff huddled around the trainer (data not shown).

### Comparison to other literature and recommendations

Complex interventions have greater scope for variation in their delivery, and so are more vulnerable to one or more components not being implemented as they should; both in delivery and actions following this [20, 41]. The fidelity of implementation model (Fig. 2) details that various moderators should be taken into consideration when implementing an intervention:

### Facilitation

Participants in this study were familiar with and positive about the use of prompts and templates. A study by Walker et al., [33] suggest that alerts alone may not be sufficient to significantly increase chlamydia screening, but that they could be included as part of a more complex intervention, such as the 3Cs and HIV. This complex intervention uses prompts in combination with hard copy resources, such as posters and leaflets. Therefore, strategies such as: providing a manual on how to set up a computer prompt based on age; or training the trainer on how to add this prompt to the system, and have the trainer set up the prompt at the first educational workshop, could facilitate implementation, which could have improved the outcomes [33].

Provision of chlamydia kits was not part of the 3Cs and HIV intervention, which relied on the use of existing kits, provided by the Clinical Commissioning Group (CCG). The chlamydia kits’ supplier was not recorded as part of the intervention, but future work may be necessary to liaise with the commissioners of primary care resources, to highlight the importance of providing the complete kits in GP surgeries, as this would resolve the problem of incomplete kits, identified by some participants. This further highlights the differences between an RCT and sustainably implementing change in real-life conditions, using existing resources and procedures.

### Intervention complexity

Research on guidelines intended for GPs found that detailed and clear recommendations were almost twice as likely to be followed as vague and non-specific recommendations [20, 43]. It could have facilitated implementation if trainers had been explicit about the need for chlamydia kits to be available in every consultation room, to facilitate staff making the offer and facilitate patients completing the tests immediately.

Additionally, more specificity was necessary in regards to where the posters should have been displayed, as the educational workshops suggested that posters displayed in consultation rooms could be used as a prompt or conversation starter, but this was not always adhered to. Furthermore, as the 3Cs and HIV is a complex intervention with multifaceted components and outcomes, a specific action plan detailing staff’s roles and responsibilities to implement the intervention should have been agreed at the end of the training session and re-visited at practice meetings [44, 45].

The lack of fidelity of implementation of the intervention may also have been a result of not everyone attending the educational workshops. The workshop highlighted that if everyone within a practice understands what the 3Cs offer is and how it works in their practice, it makes a huge difference in increasing successful outcomes [46]. Francis and Young [47] define a team as “an energetic group of people who are committed to achieving common objectives”. However, as not everyone attended the training sessions, there was rarely a whole team approach, which may have resulted in an inconsistent approach to testing, reported by participants, and thus, an overall reduction in fidelity of implementation. By: accessing existing protected learning time and networks; making attendance mandatory; or providing more regular training opportunities, the educational workshop would be attended by a higher percentage of staff, and therefore a whole team approach would be more likely.

Robbins and Finley [48] argue that team failure is often due to: mismatched needs; confused goals; unresolved roles; insufficient feedback and/or information; ill-conceived reward systems, some of which are consistent with the findings of this study.

## Conclusions

The findings of this qualitative study indicate that the overall lack of significant increases in chlamydia testing across all general practices involved in the intervention could be due to lack of adherence to the intervention as it was intended, and how it was suggested in the educational workshops. CIRT [49] highlighted key components of the chlamydia intervention that resulted in a successful and sustained increase in chlamydia screening. However, this study highlights that, although in theory these components are known, it is not always possible to implement them outside of a research setting. Further research is required to explore the differences between research and real-life settings; however, this qualitative evaluation suggests minor modifications to the presentation, and support provided within the intervention, which could greatly facilitate implementation and may increase chlamydia screening (Table 2).

**Table 2:** Programme differentiation: “key” components identified, without which, the intervention may not have its intended effect

Components of the intervention that were not implemented as intended	Importance of the component	Recommendations for improvements
Whole practice attendance at the educational workshops	If everyone understands what the 3Cs offer is and how it works in their practice, it makes a huge difference in increasing successful outcomes	Make the 3Cs and HIV training mandatory and offer more frequent educational workshops. In the meantime, continue working with the practice manager to find an appropriate time when as many people as possible can attend.
Age-based prompt as well as addition to templates	A prompt solely on the contraception template will not target young men [4] and therefore a prompt is needed for all patients aged 15–24 years old to ensure that clinicians are reminded to offer the 3Cs to everyone in this age group [33].	Be more specific in the educational workshop and facilitate by providing a manual on how to add the prompt; or train the trainer on how to add this prompt to the system, and have the trainer set up the prompt at the first educational workshop.
Complete screening kits in all clinicians’ rooms	Screening kits at the clinician’s fingertips act as a reminder and facilitates ease of testing, which saves time and will facilitate completion of the tests on-site	Be more specific in the educational workshop and ensure a detailed action plan including who makes up kits is completed in the workshop. Liaise with the commissioners to suggest provision of complete kits as this would facilitate chlamydia testing. Encourage all staff to attend the training so that the whole team: is committed; are clear on their roles; and understand the testing process in their practice.
Reminders and feedback on progress of testing and diagnosis rates	Regular feedback ensures that: 3Cs is not forgotten; staff remain focussed on any targets; and 3Cs are kept as a high priority.	Identify a sexual health champion during the educational workshop ensures that the champion is clear on their role of feeding back and discussing staff’s progress of 3Cs in team meetings. A locally enhanced service (LES) should increase the priority of 3Cs in the CCG and the GP surgery. LES financial incentives, in themselves, may not be a driver, but CCG reminders that coincide may improve chlamydia screening rates [50, 51].

The majority of practices were initially very motivated by the educational workshop, but were not able to sustain this focus, which resulted in drop off in fidelity of implementation. To counter this, it is essential that contact is sustained between the trainer and the sexual health champion identified within the educational workshop, so that any questions are answered and ensure that the suggested changes have been implemented. Although the third educational workshop was optional, the majority of practices opted for this to be carried out by phone or felt that they did not need or have time for it [19]. In an attempt to build relations, it could be advised that the trainer return to the practice to be able to see in person whether the intervention is being implemented as intended, give suggestions for improvements, and their presence would act as a reminder for staff to re-focus on their role. Although trainers attempted to find a suitable time to return to the practice, this was not always possible due to lack of time, both for the trainer who was not given additional funding or time to complete the role; and the practice who highlighted lack of time as their main barrier for implementation. It is a suggestion that practices without a locally enhanced service should be targeted first for extra support as there was not a significant increase in chlamydia testing within this group [19].

It is possible that the additional complexity of the 3Cs and HIV intervention in contrast to CIRT was not fully appreciated, and the additional support and facilitators required for implementation was not foreseen. Therefore, in an attempt to increase fidelity of implementation and consequently improve likelihood of increased screening, the intervention needs to have: more specific action planning; computer prompts added to systems and used; all staff attend the workshop in dedicated learning time; and on-going practice staff support.

## Additional files

Additional file 1: Appendix 1. Initial Interview Schedule. (DOCX 20 kb)

Additional file 2: Appendix 2. Final Interview Schedule, modified during qualitative data collection. (DOCX 20 kb)

## Abbreviations

3Cs and HIV: Chlamydia, Contraception, Condoms, HIV
CCG: Clinical Commissioning Group
CIRT: Chlamydia Intervention Randomised Trial
GP: General Practitioner
ITT: Intention to treat
LA: Local authority
LES: Local enhanced service
NCSP: National chlamydia screening programme
OSOP: One sheet of paper
PHE: Public Health England
PID: Pelvic inflammatory disease
PSHE: Personal, social, health and economic education
RCT: Randomised controlled trial
SRE: Sex and relationship education
STI: Sexually transmitted infection
