# Supplementary material for: Exploring why a complex intervention piloted in general practices did not result in an increase in chlamydia screening and diagnosis: a qualitative evaluation using the fidelity of implementation model
Source: BMC Fam Pract. 2017 Mar 21;18:43. doi: 10.1186/s12875-017-0618-0 (PMC5361828; doi:10.1186/s12875-017-0618-0)
Supplement: Supplementary file 1 — Appendix 1. Initial Interview Schedule. (DOCX 20 kb) [file 12875_2017_618_MOESM1_ESM.docx]

**Appendix 1. Initial Interview Schedule**

**Staff interview schedule – ENGLAND Post intervention V1**

**Interview information (to be read by interviewer)**

We are consulting with primary care staff to get their opinions on the 3Cs&HIV training programme their surgery has participated in which aimed to embed chlamydia screening, HIV testing and other sexual health work in primary care practices. The interviews will explore staff’s personal attitudes and the attitudes of the surgery as a whole on the training and associated resources, as well as making a broad sexual health offer and recommending an HIV test to patients according to national guidance.

We wish to record the interviews to enable us to analyse full and accurate transcripts of what was said – the data will be anonymous and confidential. We will send you a transcript if you wish in order to comment on the interview at a later stage

**RECORD:**

**Gender**

**Age**

**Practice identifier**

**Assigned number**

**HIGH/LOW Chlamydia screening practice (defined by researcher)**

**HIGH/LOW HIV prevalence (defined by researcher)**

**Date of interview**

**Name of interviewer**

**Questions**

We are interested in understanding how your own practice has been influenced, or not, since your practice participated in the training and support offered by your 3Cs&HIV trainer (establish name of trainer if possible)

**Section A - 3Cs in your practice**

1. Can you start by describing your own approach to chlamydia screening and other sexual health work before you participated in 3Cs&HIV training? (Probe: previous sexual health training, attitudes of self and other staff in practice towards sexual health of young adults)

2. What impact did attending 3C training have on you? (Probe: which intervention components did they enact- posters, cards, prompts, champion, further discussion, routine offer)

3. Were you clear on your role and that of your colleagues in taking the 3Cs forward? (coherence)

4. Did you meet as a whole practice to discuss/review how to implement the 3Cs programme? How did this go? Where there any outcomes? Probe: action planning, adjusting processes after feedback) (reflexive monitoring)

5. How easy or difficult do you find offering each of the 3 components of the 3Cs offer –a chlamydia screen, advising or signposting to contraception and free condoms? (Probe: in combination, not just separately) (perceived behavioural control)

6. How confident are you to make a 3Cs offer in consultations- what has made it easier/more difficult? (perceived behavioural control)

7. How do you think 3Cs training affected the practice of other staff around sexual health in the surgery? (Probe: which staff? If some staff not affected explore reasons) (subjective norms)

8. Has working in this way meant you, or others, have more work to do? (Probe: consultation length, maintaining screening resources)( (perceived behavioural control)

9. Have there been any changes in policies, training of staff or their roles to achieve making a routine 3Cs offer? (perceived behavioural control)

10. How do you think young people feel about receiving a 3Cs offer? (Probe: different age groups, male/female patients) (personal attitude)

11. How did you think young people felt about completing the test there and then? Did this work in practice? (cognitive participation/collective action)

12. What affect has 3Cs training/working in this way had on the relationship you have with patients? (probe: does this vary in different types of consultations) (personal attitude)

13. How would you evaluate the 3Cs training and support? (Probe: balance of info between CT, contraception and condoms, more detail, more frequent contact from trainer, other information) (personal attitude)

14. What impact did the 3Cs training have on the practice’s chlamydia testing processes in your practice? Did anything change following the training? (collective action/reflexive monitoring)

15. Did you use computer prompts to remind staff to offer the test during consultations? (Probe: if so, how perceived/useful? If not, why not?)

16. Do you know how/whether your testing rates and diagnoses have changed? (Probe: access to data, interest in monitoring) (cognitive participation/reflexive monitoring)

**Section B - HIV testing in practice**

1. Was there an HIV training session run at your practice? (If not, explore why. Describe training content and ask whether they would have liked to receive it)

2. Did you attend the HIV training session? (Probe: If not, did other staff, and with what impact? If no HIV training, explore why not)

3. Can you describe first your own approach and that of others in the surgery to HIV testing before the training?

4. What impact did the HIV training at your practice have on your own practice and that of others? (Probe: new patient registrant testing feasibility, recommending test on indicator illness. Have they offered a test? Which resources did the practice implement - posters, prompts, script ideas etc.)

5. When did you last carry out an HIV test for a patient? (Probe: on what basis did you offer the test? Which test did you use? What was the result? What did you do then?)

6. What do you think other staff here feel about undertaking HIV testing in the surgery?) (Probe on what basis testing occurs. Which staff are testing? Is testing discussed?) (subjective norms)

7. Has your surgery had any positive results for HIV tests (ever)?

8. How do you think patients feel about being recommended an HIV test in your practice? (Probe: different age groups, male/female patients, at risk groups) (Personal attitudes)

9. How would you evaluate the HIV training and support? (Probe: more/less/different information (type?), other resources, more support from trainer) (Personal attitudes)

**Section C - Data entry and collection**

**1.** Have you undertaken data searches for your 3Cs&HIV work? (Probe: LARC data, HIV testing data. On what basis – surgery audit or to give trainer? If not, how easy/difficult would that be. ) (perceived behavioural control)

**Section D Future sexual health work**

**2.** Can you describe what further input from a trainer you would like to support sexual health work in your surgery in the future? (Probe: training in surgery, newsletters, e-learning, which topics) (Personal attitude)

**Section E Information about the surgery, practitioner and local attitudes to sexual health of young adults**

**1.** Could you tell me about your surgery? (Probe: What are the characteristics of this surgery (e.g. training, R&D, ethnic groups do you serve?)

2. What are your views on the importance of the young adults’ sexual health to commissioners in this area? (subjective norms)

3. What impact do you feel the changes in the organisation of the NHS and public health locally have had on the delivery of sexual health services in your surgery? (perceived behavioural control)

Summarise key points of the discussion and invite final comments

Thank you very much for participating.
